# Genetic Effects of Vascular Endothelial Growth Factor A (VEGF-A) and Its Association with Disease Progression in Breast Cancer Population of Saudi Arabia

**DOI:** 10.31557/APJCP.2020.21.1.139

**Published:** 2020

**Authors:** Ibrahim Altedlawi Albalawi, Rashid Mir, FM Abu Duhier

**Affiliations:** 1 *Department of Surgical Oncology, Faculty of Medicine, *; 2 *Prince Fahd Bin Sultan Research Chair, Department of Medical Laboratory Technology, Faculty of Applied Medical Sciences, University of Tabuk, Kingdom of Saudi Arabia. *

**Keywords:** Breast cancer, angiogenesis, vascular endothelial growth factor A, metastasis, gene polymorphisms Cancer

## Abstract

**Aim:**

Previous studies have shown that vascular endothelial growth factor (VEGFA) gene variants were associated with breast cancer risk. The goal of the current study was to evaluate the genetic effects of the vascular endothelial growth factor (VEGF) on the risk of breast cancer and its association with disease progression.

**Methodology:**

This case control study was conducted on 110 Breast cancer cases and 110 gender matched healthy controls. Vascular endothelial growth factor A (VEGF-A) 1 (-460T>C) genotyping was performed using Amplification refractory mutation system PCR method. The vascular endothelial growth factor A (VEGF-A) (-460T>C) genotypes were collated with different clinicopathological features of breast cancer patients.

**Results:**

A significant difference was observed between the genotype distribution of VEGF-A (-460T>C) among breast cancer cases and gender matched healthy controls (p=0.006). The frequencies of all three genotypes CC,CT,TT reported in the breast cancer patients and sex matched healthy controls were 4.54%, 46.36% ,49.20% and 7.27%, 64.54%, 28.18% respectively. The increased susceptibility to breast cancer disease was found to be associated with VEGF (-460T>C) CC vs TT variant in codominant inheritance model OR 2.78 (0.83-9.26) RR 1.68(1.01 to 2.81) P=0.04. A significant association was reported with VEGF (-460T>C) (CC+CT vs. TT) variant in recessive inheritance model, (OR=2.45 (95% CI= (1.40-4.29), P=0.003. Our findings indicated that VEGF (-460T>C) TT genotype is associated with an increased susceptibility to breast cancer disease. Our result indicates a potential dominant effect of VEGF (-460T>C) TT genotype on susceptibility to the breast cancer disease.

**Conclusion:**

VEGF (-460T>C) TT genotype significantly increased the risk of breast cancer. VEGF-A (-460T>C) genetic ariability was significantly associated with distant metastasis of the disease. It may be a useful as predisposing genetic marker for breast cancer .Further studies with larger sample sizes are necessary to confirm our findings.

## Introduction

Breast cancer is the most common malignancy in women around the world and one in 12 women in the West develops breast cancer at some point in life. About 5-10% of all breast cancer cases in women are associated to hereditary susceptibility due to mutations in autosomal dominant genes (Bray et al., 2013). The Breast cancer malignancy carries tremendous socio-economic, emotional, and public health implications. Recently a significant increase in the incidence of breast cancer has been reported in this part of globe (Saudi Arabia), which occurs at an earlier age than in western countries. Despite this finding, low participation rates in BC screening activities have been reported among Arab women (Al Balawi et al., 2018; Mir et al., 2018). Most of the women, regardless of their ethnic origin or racial or heritage are at risk of developing breast cancer. The variations in breast carcinoma incidence rates among multicultural populations suggest that etiologic factors differ in their biologic expression and impact on disease outcome. The key among those factors that affect breast carcinoma development are the roles of genetics and the environment, the reproductive experience and the effects of endogenous and exogenous hormones in women, the change in immune status and host vulnerability, and the biologic determinants of breast carcinoma (Lee et al., 2018). Molecular studies have increased the understanding of the mechanisms of chemotherapy and hormone resistance, such as the role of mutations in estrogen and progesterone receptor in resistance to endocrine therapy (Cheon et al., 2002). 

Aberrant angiogenesis is one of the most studied genetic alterations in cancer development. In fact, angiogenesis is a critical process in the development, growth, and metastasis of malignant tumors. The overexpression of vascular endothelial growth factor (VEGF), the key mediator of angiogenesis, is associated with poor prognosis in cancer (Nieder et al., 2003). The VEGF gene is located on chromosome 6p12-p21, and consists of eight exons separated by seven introns that exhibit alternative splicing to form a family of proteins. Recently the inhibition of angiogenesis has demonstrated clinically significant improvements in outcomes in a variety of malignancies, including breast cancer. During the past 2 decades, Bevacizumab, an agent recognizing and blocking the pathway of VEGF-A, has shown some efficacy in combination regimens to improving progression-free survival and overall survival of some solid malignancies, such as breast cancer, renal cell carcinoma, and non-small-cell lung cancer (NSCLC) (Welch et al., 2010).

Previous studies confirmed that VEGF gene has at least 30 single-nucleotide polymorphisms. The expression of *VEGFA* gene is highly modulated, and single nucleotide polymorphisms occur in the promoter region (rs699947, rs1570360 and rs833061), in the 5’-untranslated region (rs2010963, rs25648) and in the 3’untranslated region (rs3025039, rs10434), next to many potential regulatory elements (Brogan et al., 1999). Among them, –2578C>A (rs699947), –634G>C (rs2010963), –460C>T (rs833061), and +936C>T (rs3025039) were demonstrated to regulate VEGF expression (Ruiz et al., 2012; Chung and Chanock, 2011) VEGFA SNPs were shown to affect VEGFA levels in different cell models, (Watson et al., 2000) including breast cancer (Krippl et al., 2003). *VEGFA* gene polymorphisms have also been associated with breast cancer susceptibility, (Lu et al., 2005) increased risk of disease progression (Absenger et al., 2013) or poor survival (Kidd et al., 2010). Therefore, genetic variability of the *VEGF *gene could also modulate breast carcinogenesis through affecting angiogenesis. One *VEGF* gene polymorphism (rs833061) located in the promoter region of the *VEGF *gene, reported to be important that might influence the promoter activity. High VEGF expression are associated enhanced tumor vasculature might facilitate the delivery of chemotherapy agents to target tumor issues and also inhibit tumor radioresistance caused by radiation-induced hypoxia, leading to a better synergistic effect between chemotherapy and radiotherapy. However, the results of previous studies remain inconclusive and controversial. The goal of the current study was to evaluate the genetic effects of the vascular endothelial growth factor A (VEGFA) (-460T>C) on the risk of Breast cancer and its association with disease progression. 

## Materials and Methods

This population-based case–control study was conducted on 110 histologically confirmed breast cancer patients and 110 gender matched healthy women with no history of any types of cancer and were not related to the patients. 

*Clinical Data*

Both patients as well as healthy controls were interviewed using a structured questionnaire regarding epidemiological/demographic data, past history, family history of cases. The detailed laboratory and clinical data were collected to determine relevant clinical history. This research was approved by the Research Ethics Committee, University of Tabuk and a written informed consent was obtained from all the subjects before enrollment. 

*Sample collection*

Peripheral blood samples from breast cancer patients as well as from gender matched healthy controls were collected by an experienced lab technologist. After assessing the clinicopathological findings, a 3ml sample of peripheral blood was collected by venipuncture in EDTA tubes. Patients with any previous history of cancer were excluded from this study.

*DNA extraction from peripheral blood of cases and controls*

Peripheral blood samples were transported from different hospitals (Cooling containers) to molecular biology laboratory of Prince Fahd Bin Sultan Research chair. Blood samples were processed for genomic DNA extraction using DNeasy Blood Kit (cat 69506) from Qiagen (Germany) as per the manufactures instructions. The extracted DNA was dissolved in nuclease-free water and stored at 4°C until use. Quality and integrity of DNA were checked by NanoDrop™ (Thermo Scientific, USA).

*VEGF-A polymorphism rs833061 (-460T>C)*

Vascular endothelial growth factor A (VEGF-A) (-460T>C) genotyping was performed using Amplification refractory mutation system PCR method -ARMS-PCR. ARM Systems are based on the use of sequence-specific PCR primers that allow amplification of test DNA only when the target allele is contained within the sample. Following an ARMS reaction the presence or absence of a PCR product is diagnostic for the presence or absence of the target allele. The VEGFA (-460T>C) genotyping primers were designed by using primer3 software as indicated in Table 1.

The ARMS-PCR was performed in a reaction volume of 25uL containing template DNA (50ng), FO -0.30uL, RO -0.30uL , RI -0.20uL , RI -0.20uL of 25pmol of each primers and 10uL from GoTaq^® ^Green Master Mix (cat no M7122) (Promega, USA). The final volume of 25 uL was adjusted by adding nuclease free DEPC H2O. Finally, the 2ul of DNA was added from each patient. 

*Thermocycling conditions*

**Figure 1:**
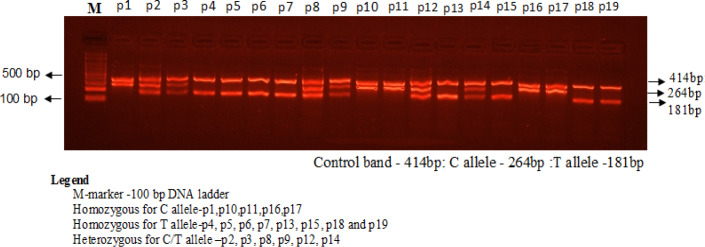
Detection of VEGF-A Polymorphism (-460T>C) by Using Amplification-Refractory Mutation System –PCR (ARMS-PCR) in Breast Cancer Patients

The amplification conditions used were at 95^o^C for 10 minutes followed by 40 cycles of 94^o^C for 35 sec, 56^o^C for 40 sec, 72^o^C for 45 sec followed by the final extension at 72^o^C for 10 minutes. Optimization was done by performing gradient PCR .The best temperature was determined in the temperature range of 56°C to 64°C tested with a gradient PCR thermocycler. The number of cycles was increased from 35 to 43 cycles, significantly enhancing the yields of all three PCR products. Together, these changes resulted in a more robust amplification of the mutant allele and a less competing reaction from the control, as shown by the relative intensities of the corresponding bands on agarose gel electrophoresis as depicted in Figure 1. The annealing temperature was lowered from 60 to 56°C to favor the binding of both forward wild and reverse mutant primers that contain mismatches to the templates.

*Gel electrophoresis*

The amplification products were separated by electrophoresis through 2% agarose gel stained with 0.5μg/mL ethidium bromide and visualized on a UV transilluminator. Primers FO and RO flank the exon of the VEGFA gene resulting a band of 414bp to act as a control for DNA quality and quantity. Primers Fwt and RO amplify a wild-type allele (C allele), generating a band of 264bp, and primers FO and Rmt generate a band of 181bp from the mutant allele (T allele) as indicated in Figure 1.

*Statistical analysis*

The associations between VEGF-A (-460T>C), genotypes and risk of breast cancer were estimated by computing the odds ratios (ORs), risk ratios (RRs) and risk differences (RDs) with 95 % confidence intervals (CIs). Differences in the VEGF-A (-460T>C), gene allele and genotype frequencies between groups were evaluated using Chi-square test. Group differences were compared using Student’s two-sample t-test or one-way analysis of variance (ANOVA) for continuous variables and Chi-squared for categorical variables. 

## Results

The Hardy-Weinberg Equilibrium Analysis: The genotype distributions and allele frequencies of VEGF-A rs833061 (-460T>C) gene was in strong linkage disequilibrium as detected in HWE (all p > 0.05) (χ^2^ = 0.44 P=0.612) in the patient group and in control group. Thus, we chose 10% samples from gender matched healthy control group randomly to review genotyping results, showing that the accuracy rate was more than 99%. 

*Study Population*

This population-based case–control study was conducted on 220 subjects among whom 110 were histologically confirmed breast cancer patients and 110 were gender matched healthy women with no history of any type of cancer. 

*Demographical Profile of the Enrolled breast Cancer Patients*

The clinicopathological characteristics of breast cancer patients are shown in Table 3. Of 110 consecutive breast cancer patients, 40 (26.37%) were below or equal to 40 years age and 70 (63.63%) were above 40 years of age. Of breast cancer cases 30 (31.82%) were in early (I and II) stage and 70 (68.18%) cases were in advanced stages (III and IV). Histological grading of the patient tumor showed that 10 (9%), 32 (29%) and 58 (61.81%) were in grade I, II and III respectively. Out of 110 cases, 78 (70.90%) patients had distant metastasis and 32 (29.10%) does not show distant metastasis. Based on the receptor status, out of 110 Breast cancer cases 45 (40.90%) were positive for Her2/neu, 62 (56.37%) were carrying estrogen receptor and 72 (64.45%) were +ve for progesterone receptor. Out of 110 cases, 30(27.27%) patients were treated with herceptin and 80 (72.73%) were not treated with herceptin. Tamoxifen has been the basis of endocrine therapy for patients with ER (+) breast cancer for more than three decades. The treatment reduces the annual mortality rate of breast cancer by 31%, and remains the most effective targeted cancer therapy. However, approximately one-third of patients treated with adjuvant Tamoxifen suffer from aggressive recurrent disease. Resistance to tamoxifen, thus, remains a major challenge in providing effective treatments for these patients. Out of 110 cases, 67 (60.90%) patients were treated with Tamoxifen and 43 (29.10%) were not treated with Tamoxifen. There are many conflicting results in the literature comparing quality of life following breast-conserving therapy (BCT) and mastectomy. In our study, out of 110 cases, 33 (30%) patients received mastectomy and 77 (70%) did not received mastectomy. Lumpectomy (also known as breast conserving surgery, partial mastectomy or wide excision) is a surgery to remove cancer from the breast. Unlike a mastectomy, a lumpectomy removes only the tumor and a small rim of normal tissue around it. It leaves most of the breast skin and tissue in place. In our study, out of 110 cases, 24 (21.81%) patients received Lumpectomy and 86 (78.18%) did not received Lumpectomy.

*Association of VEGF-A rs833061 (-460T>C) between Cases and Controls*

We observed a statistically significant difference in the frequencies of VEGF-A rs833061 (-460T>C) between the Breast cancer patients and gender matched healthy controls (p=0.006) as indicated in Table 4. The frequencies of the genotypes CC, CT, TT in breast cancer patients were 4.54%, 46.36% and 49.20%, and in healthy controls were 7.27%, 64.54% and 28.18% respectively .The frequency of VEGF-A rs833061 T allele was found to be higher among breast cancer patients (0.72) than the healthy controls (0.60). 

*Correlation of VEGF-A (-460T>C) with respect to clinicopathological features*

This study observed that VEGF-A (-460T>C) variants TT genotype was associated with increased breast cancer risk (P= 0.006). A non-significant correction was observed between VEGF-A (-460T>C) genotypes with respect to histological grades, stage, status however a statistically significant association was reported between the VEGF-A (-460T>C) genotypes with respect to the distant metastasis of the disease (P<0.03) as depicted in Table 5. A non-significant correlation was reported between VEGF-A (-460T>C) genotypes in breast cancer patients with age status (p=0.57). Similarly a non-significant correction was revealed between VEGF-A (-460T>C) genotypes of breast cancer cases with estrogen receptor as well as progesterone receptor status (P=0.63 and 0.34) whereas a significant correction was revealed with respect to her2/neu receptor status (P=0.001) as depicted in Table 5. A significant correlation between VEGF-A (-460T>C) genotypes in breast cancer patients with respect Tamoxifen treatment was revealed (p=0.005) and also a significant association of VEGF-A (-460T>C) genotypes in breast cancer patients with Trastuzumab or Herceptin treatment was reported (p=0.026).

*Correlation between VEGF-A (-460T>C) gene variability and breast cancer risk*

A multivariate analysis based on logistic regression like odds ratio (OD) and risk ratio (RR) with 95% confidence intervals (CI) was calculated for each group to estimate the association between VEGF-A (-460T>C) genotypes and risk to breast cancer disease as indicated in Table 6. Our result indicates a potential dominant effect of VEGF-A (-460T>C) TT genotype on susceptibility to the breast cancer disease. The increased susceptibility to breast cancer disease in Saudi Arabian population was found to be associated with VEGF-A (-460T>C) CC vs TT variant in codominant inheritance model, OR 2.78 (0.83-9.26) RR 1.68 (1.01 to 2.81) P=0.04. A significant association was reported with VEGF-A (-460T>C) (CC+CT vs. TT) variant in recessive inheritance model, (OR=2.45 (95% CI= (1.40-4.29), P=0.003. 

**Table 1:** Amplification-Refractory Mutation System –PCR primers for VEGF-A rs833061 (-460T>C) Gene Polymorphism

Direction	Primer sequence	PCR product size	AT
Fo *VEGF-A*	5-CAAAGCCCATTCCCTCTTTA-3	414bp	56
Ro *VEGF-A*	5-CACAGCCTGAAAATTACCCA-3		
FI C* VEGF-A*	5-CGTGTGGGGTTGAGTGC-3	264bp	
RI T *VEGF-A*	5-CTCCCCGCTCCACCA-3	181bp	

Fo-outer forward primer, Ro-Reverse outer primer; AT, annealing temperature; FI-Inner, forward primer; RI-Inner, Reverse primer.

**Table 2:** Preparation of PCR Cocktail for VEGF-A rs833061 (-460T>C)genotyping

	1x
PCR master mix	10ul
Forward primer FO	0.30 ul
Reverse primer RO	0.30 ul
Forward primer FI T	0.20 ul
Reverse primer RI C	0.20 ul
Nuclease free water	12ul
Total volume	23ul
DNA (50ng)	2ul

**Table 3:** Clinicopathological Characteristics of Breast Cancer Patients

Parameters	110	
Age Group		
Age >40	70	63.63%
Age<40	40	26.37%
Stage status		
Early (I and II)	35	31.82%
Advanced (III and IV)	75	68.18%
Grade I vs Grade II		
Grade I	10	9.09%
Grade II	32	29.00%
Grade III	68	61.81%
Estrogen receptor status		
Positive	62	56.37%
Negative	48	43.63%
Progesterone Receptor status		
Positive	72	65.45%
Negative	38	34.55%
Her2/neu status		
Positive	45	40.90%
Negative	65	59.10%
Distant metastasis status		
Positive	78	70.90%
Negative	32	29.10%
Herceptin treatment		
Herceptin	30	27.27%
No Herceptin	80	72.73%
Tamoxifen treatment		
Tamoxifen	67	60.90%
No Tamoxifen	43	39.10%
Mastectomy treatment		
Mastectomy	33	30.00%
No Mastectomy	77	70.00%
Lumpectomy Surgery		
Lumpectomy	24	21.81%
No Lumpectomy	86	78.18%

**Table 4:** Association of *VEGF-A* rs833061 (-460T>C) Gene Variability between Cases and Controls

Subjects	N=	CC	CT	TT	C allele	T allele	X^2^	P-value
Breast cancer	110	5 (4.54%)	51 (46.36%)	54 (49.20%)	0.28	0.72	10	0.006
Healthy Controls	110	8 (7.27%)	71 (64.54%)	31 (28.18%)	0.4	0.6		
Total	220	13	122	85				

**Table 5:** Correlation between *VEGF-A* rs833061 (-460T>C) and Clinicopathological Characteristics of Breast Cancer (BC) Patients

Parameters	110	CC	CT	TT	X^2^	Df	P-value
Age Group							
Age >40	70	3	30	37	1.1	2	0.57
Age<40	40	2	21	17			
Stage status							
Early (I and II)	35	2	21	12	4.51	2	0.1
Advanced (III and IV)	75	3	30	42			
Grade I vs Grade II							
Grade I	10	1	5	4	4.39	2	0.11
Grade II	32	2	6	24			
Grade I	10	1	5	4	1.25	2	0.53
Grade III	68	2	40	26			
Estrogen receptor status							
Positive	62	3	31	28	0.88	2	0.64
Negative	48	2	20	26			
Progesterone Receptor status							
Positive	72	2	36	34	2.18	2	0.33
Negative	38	3	15	20			
Her2/neu status							
Positive	45	3	6	36	33.49	2	0.0001
Negative	65	2	45	18			
Distant metastasis status		CC	CT	TT			
Positive	78	3	45	30	13.88	2	0.001
Negative	32	2	6	24			
Herceptin treatment							
Herceptin	30	1	8	21	7.26	2	0.026
No Herceptin	80	4	43	33			
Tamoxifen treatment							
Tamoxifen	67	2	41	24	15.2	2	0.005
No Tamoxifen	43	3	10	30			
Mastectomy treatment							
Mastectomy	33	3	16	14	2.62	2	0.26
No Mastectomy	77	2	35	40			
Lumpectomy Surgery							
Lumpectomy	24	3	11	10	4.62	2	0.9
No Lumpectomy	86	2	40	44			

**Table 6:** Multivariate Analysis to Study Correlation between *VEGF-A* Rs833061 (-460T>C) Gene Variability and Breast Cancer Risk

Genotypes	Healthy Controls (N=110)	Breast Cancer Patients (N=110)	OR (95% CI)	Risk Ratio (RR)	P-Value
Codominant					
VEGFA-CC	8	5	1 (ref.)	1 (ref.)	
VEGFA-CT	71	51	1.14 (0.35 to 3.71)	1.05 (0.67-1.66)	0.81
VEGFA-TT	31	54	2.78 (0.83-9.26)	1.68 (1.01 to 2.81)	0.04
Dominant					
VEGFA-CC	8	5	1 (ref.)	1 (ref.)	
VEGFA-(CT+TT)	102	105	1.64 (0.52-5.21)	1.24 (0.79-1.96)	0.39
Recessive					
VEGFA-(CC+CT)	79	56	1 (ref.)	1 (ref.)	
VEGFA-TT	31	54	2.45 (1.40-4.29)	1.60 (1.17 to 2.19)	0.003
Allele					
VEGFA-C	87	61	1 (ref.)	1 (ref.)	
VEGFA-T	133	110	0.77 (0.49 to 1.20)	0.88 (0.70- 1.09)	0.26

## Discussion

The goal of the current study was to evaluate the genetic effects of the vascular endothelial growth factor A VEGF-A (-460T>C) on the risk of breast cancer and its association with disease progression. Recently, the associations between VEGF-A (-460T>C) polymorphisms and the risk of breast cancer have been extensively studied; however, the reported results have been inconsistent. The lack of concordance across many of these studies reflects their limitations including small sample sizes, ethnic differences, and research methodologies. The study was conducted in a prospective manner, based on a multiinstitution cohort of Saudi Arabian women with breast cancer. However relatively limited in the sample size, the study design allowed full verification of the patients’ clinical history, as well as minimization of diversity in therapeutic conducts and in follow-up routines. In the current study, VEGF-A (-460T>C) genetic variability was significantly associated with stage as well as distant metastasis of the disease. Stevens et al ,2003 (Stevens et al., 2003) observed that C variants of VEGF −460C>T were associated with increased VEGF promoter activity and therefore it may be possible that VEGF –460 C allele can enhance the tumor angiogenesis. 

In case of breast cancer patients who are treated with chemotherapy and radiotherapy may enhance tumor vasculature that can facilitate the delivery of chemotherapy agents to target tumor issues and also inhibit tumor radioresistance caused by radiation-induced hypoxia, leading to a better synergistic effect between chemotherapy and radiotherapy. Our results were in accordance with the previous studies conducted by Cao et al., (2011) and Wang et al., (2011) in their meta-analyses reported that *VEGFA–460C>T* gene variation was related to risk of breast cancer ,gastric cancer and colorectal cancer.

Our study reported a significant association of VEGF-A (-460T>C) with distant metastasis with higher lymph node invasion, suggests that this SNP of VEGFA possibly either segregate with more aggressive breast cancer phenotypes or may lead to higher VEGF levels, which is known to be associated with worse outcomes of breast cancer similar association was reported by Gasparini et al., (1997) and Gasparini, (2000) similarly Radovich et al., (2010) evaluated the impact of VEGFA haplotypes in gene reporter systems, that indicated that the two haplotypes containing the alleles A of rs833061 and C of rs699947 induced higher luciferase expression in most cell lines, both in hypoxia as well as in normoxia conditions. 

Also Koukourakis et al., (2004) reported increased VEGF expression for theT allele of VEGF-A (-460T>C) polymorphisms. Pharmacogenomic studies on angiogenesis inhibitors are currently somewhat rare. However, one group Schneider et al., (2008) has identified an association between VEGF promoter polymorphisms and breast cancer response to Bevicuzimab, with genotypes consistent with haplotype 4 associated with dramatic extension of overall survival similarly in our a significant correlation between VEGF-A rs833061 genotypes in breast cancer patients with respect Tamoxifen treatment was revealed (p=0.005) and also a significant association of VEGF-A (-460T>C) genotypes in breast cancer patients with Trastuzumab or herceptin treatment was reported (p=0.03). Because angiogenesis is a critical step in the establishment and pathogenesis of breast cancer, this process has been viewed as a potential new target to better define the mechanisms that cause the carcinogenesis. A large number of studies have observed that VEGF was significantly higher in women with advanced breast cancer, which supported a key role for VEGF in the pathological angiogenesis in breast cancer. The discrepancies between different research studies involving the impact of *VEGF* gene polymorphisms on the susceptibility to breast carcinogenesis may be caused by different allele frequencies and heterogeneity in the study populations, besides environmental backgrounds. 

Nowadays, it is becoming increasingly important to derive data from different populations to build a database which can then be used for future investigations to a better understanding of the genetic and environmental factors affecting risk to development breast cancer. Our research results indicated that VEGF (-460T>C) gene variation may be a useful as predisposing genetic marker for breast cancer .Further studies with larger sample sizes are necessary to confirm our findings.

In conclusion, VEGF (-460T>C) TT genotype significantly increased the risk of breast cancer. VEGF-A (-460T>C) genetic variability was significantly associated with distant metastasis of the disease. It may be useful as a predisposing genetic marker for breast cancer. Further studies with larger sample sizes are necessary to confirm our findings. 

## Abbreviation

VEGFA- vascular endothelial growth factor A , ARMS-Amplification refractory mutation system, OR-Odds ratio, CI-Confidence interval.
